# Genomic and transcriptomic analyses reveal differential regulation of diverse terpenoid and polyketides secondary metabolites in *Hericium erinaceus*

**DOI:** 10.1038/s41598-017-10376-0

**Published:** 2017-08-31

**Authors:** Juan Chen, Xu Zeng, Yan Long Yang, Yong Mei Xing, Qi Zhang, Jia Mei Li, Ke Ma, Hong Wei Liu, Shun Xing Guo

**Affiliations:** 1 Key Laboratory of Bioactive Substances and Resource Utilization of Chinese Herbal Medicine, Ministry of Education, Institute of Medicinal Plant Development, Chinese Academy of Medical Sciences & Peking Union Medical College, Beijing, 100193 P. R. China; 2 State Key Laboratory of Mycology, Institute of Microbiology, Chinese Academy of Sciences, Beijing, 100101 P. R. China

## Abstract

The lion’s mane mushroom *Hericium erinaceus* is a famous traditional medicinal fungus credited with anti-dementia activity and a producer of cyathane diterpenoid natural products (erinacines) useful against nervous system diseases. To date, few studies have explored the biosynthesis of these compounds, although their chemical synthesis is known. Here, we report the first genome and tanscriptome sequence of the medicinal fungus *H. erinaceus*. The size of the genome is 39.35 Mb, containing 9895 gene models. The genome of *H*. *erinaceus* reveals diverse enzymes and a large family of cytochrome P450 (CYP) proteins involved in the biosynthesis of terpenoid backbones, diterpenoids, sesquiterpenes and polyketides. Three gene clusters related to terpene biosynthesis and one gene cluster for polyketides biosynthesis (PKS) were predicted. Genes involved in terpenoid biosynthesis were generally upregulated in mycelia, while the PKS gene was upregulated in the fruiting body. Comparative genome analysis of 42 fungal species of Basidiomycota revealed that most edible and medicinal mushroom show many more gene clusters involved in terpenoid and polyketide biosynthesis compared to the pathogenic fungi. None of the gene clusters for terpenoid or polyketide biosynthesis were predicted in the poisonous mushroom *Amanita muscaria*. Our findings may facilitate future discovery and biosynthesis of bioactive secondary metabolites from *H. erinaceus* and provide fundamental information for exploring the secondary metabolites in other Basidiomycetes.

## Introduction

The lion’s mane mushroom, *Hericium erinaceus* (Russulales, Hericiaceae) has a long history of use as a famous traditional Chinese medicinal fungus in Eastern Asia. *H. erinaceus* can produce unusual pharmacological effects including anti-aging, antioxidant, anti-tumor and even anti-dementia activity^1^. Thus, it has potential applications for the prevention of many age-associated neurological dysfunctions, including Alzheimer’s disease (AD) and Parkinson’s disease^2^. In particular, erinacines and hericenones, the two specific classes of natural compounds isolated from *H. erinaceus*, have been reported to stimulate nerve growth factor (NGF) synthesis in cultured astrocytes^3, 4^. Thus, the increasing demand for this mushroom for clinical experiments and functional foods has attracted considerable attention from researchers^5^.

Studies on the chemical isolation and physiological functions of *H. erinaceus* have progressed in recent years. A large number of structurally different bioactive compounds were isolated from the fruiting bodies and the mycelia of *H. erinaceus*. Apart from primary polysaccharide compounds^6^, more than 80 kinds of bioactive secondary metabolites with antitumor, antibacterial, hypoglycemic and neuroprotective effects were isolated from the free-living mycelium (FLM) or fruiting body (FB) of *H. erinaceus* ^1^. These secondary metabolites mainly included terpenoids (erinacines)^7–10^, phenols (hericenone A~E)^11^, pyrones (erinapyrones A~C)^12^, sterols (erinarol, hericerins, hericenes)^13–16^, fatty acids, volatile aroma compounds^17^ and non-ribosomal peptides (fumitremorgin C)^13^. Among them, the erinacines, cyathane-type diterpenoids with potential neuroprotective properties, have attracted considerable interest as unique bioactive secondary components of *H. erinaceus*. It has been established that approximately 20 of the 25 diterpenoids isolated from the FLM of *H. erinaceus* belong to the cyathane-type diterpenoid group^1^, such as erinacines A-I, P, Q and R^18^. They are all unified by the presence of a characteristic 5-6-7 tricarbocyclic fused core structure and are essentially a D-xylose moiety anchored onto the cyathane framework, representing as interesting target compounds for fungal biotechnology^19^. The biosynthetic pathway involved in synthesis of the cyathane core tricycle involves cascade cyclization and subsequent rearrangement of geranylgeranyl phosphate^19^. Although the chemical synthesis of bioactive compounds of cyathane-type diterpenes has been elucidated^20^, their biosynthetic pathway and gene regulation are unknown.

Here, the genome and transcriptome of *H. erinaceus* were sequenced for the first time. We aimed to i) characterize the genome of *H. erinaceus* to understand its gene content and genomic structure; ii) identify the gene clusters associated with bioactive secondary metabolites, such as terpenoid and polypeptide biosynthesis, and iii) profile gene expression differences across three different tissues: the monokaryotic mycelium (MK) and dikaryotic mycelium (DK) and fruiting body (FB) of *H. erinaceus*. The *H. erinaceus* genome sequence will provide insights into biosynthetic pathways for bioactive secondary compounds and fundamental information for improving compound production.

## Results

### Genome sequencing and feature

We sequenced a single-nucleated genomic DNA sample of strain Herl to obtain the genome using MPS (massively parallel sequencing) Illumina technology (Supplementary Fig. S1). The genome of strain Herl was assembled into 519 scaffolds with an N50 of 538 kb and a total genome size of 39.35 Mb with approximately 200-fold sequencing depth and approximately 302-fold genome coverage (Table 1, Supplementary Table S1). In total, 9895 gene models were predicted, with an average sequence length of 1345 bp. The genome size and number of predicted genes are consistent with other medicinal mushroom (Table 1). Functional annotation of the predicted genes was performed using BLAST^21^ against the following six databases: Gene ontology (GO)^22^, Kyoto Encyclopedia of Genes and Genomes (KEGG)^23^, Clusters of Orthologous Groups (COG)^24^, Evolutionary Genealogy of Genes: Non-supervised Orthologous Groups (EggNOG)^25^, Swiss-Prot^26^ and NCBI non-redundant (nr) protein database (Supplementary Table S2). In summary, we obtained annotations for 7112 and 7267 of the 9895 genes in the nr and eggNOG databases, respectively. In addition, 581 genes were annotated as secretory proteins.

**Table1:** Genomic features of *H. erinaceus* and other 6 selected medicinal fungi

	***Hericium erinaceus***	***Antrodia cinnamomea***	***Ganodema lucidum***	***Cordyceps militaris***	***Tremella mesenterica***	***Wolfiporia cocos***	***Inonotus baumii***
Accession no.	PRJN361338	JNBV00000000	AGAX00000000	AEVU00000000	AFVY00000000	AEHD00000000	LNZH00000000
Genome size (MB)	39.35	32	43.3	32.2	28.64	50.48	31.64
Genome Coverage	200x	878x	440x	147x	7.4x	40.48x	186x
Scaffold	519	360	82	32	45	348	217
Contigs	1118	648	194	597	484	1625	339
G+C content	53.14	50.6	59.3	51.4	46.7	52	47.6
Protein-coding genes	9895	9254	16113	9684	8309	12746	8455
Sequencing method	Illumina Hiseq	Roche 454; Illumina GAIIx	Roche 454	Roche 454 GS FLX system	Sanger	Sanger;454;Illumina	Illumina HiSeq

Gene ontology (GO) analysis showed that 1414, 1381, and 1225 genes were annotated with the three main categories of molecular function, cellular component and biological process, respectively (Supplementary Fig. S2). KEGG analysis showed that the highest number of genes of *H. erinaceus* were involved in metabolic processes. Carbohydrate metabolism contained the highest gene number (479 genes), followed by amino acid metabolism (405 genes) and xenobiotic biodegradation (313 genes). Sixty genes were found in terpenoid and polyketide biosynthesis (Supplementary Fig. S2).

### Transcriptomic analysis and gene expression differences

**Figure 1:**
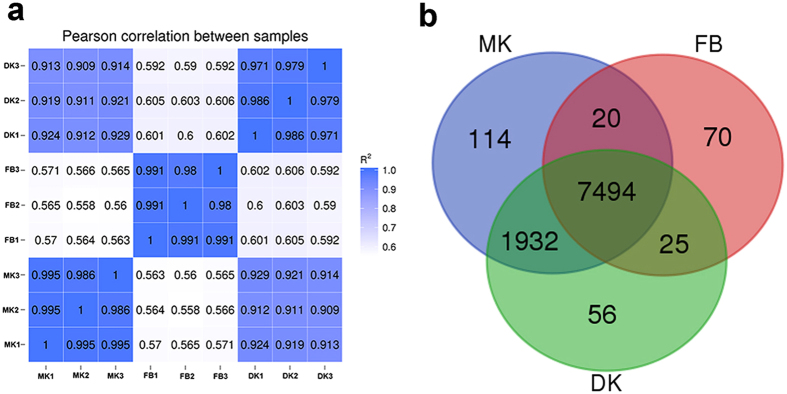
Relationships among three transcriptomes of *Hericium erinaceus*. (**a**) Pairwise correlation of normalized FPKMs between RNA samples. The Pearson correlation coefficient ranges from no correlation (white) to perfect correlation (dark blue). (**b**) Venn diagram of 9711 expressed genes in three different tissues of *H. erinaceus*. Abbreviation: MK, monokaryotic mycelium; DK, dikaryotic mycelium; FB, fruiting body.

We studied the gene expression differences between the transcriptome of MK, DK and FB of *H. erinaceus*. Three biological replicates were performed for each tissue. As showed in Fig. 1, the expression variation was the smallest between the MK and DK and greatest between the MK and FB. Similarly, the highest expression correlation occurred between the DK and MK. Among 9895 gene models in the genome of *H. erinaceus*, 9711 genes models were expressed across three different tissues (MK, DK and FB), 7494 genes were commonly expressed in all three tissues and 114, 56 and 70 genes were specifically expressed in the MK, DK and FB, respectively (Fig. 1).

Tissue-specific expression transcripts in *H. erinaceus* are listed in Supplementary Table S3. Gene encoding manganese-dependent peroxidase (MnP), heat shock protein, phosphatidylethanolamine binding protein, nitrosoguanidine resistance protein and cytochrome P450 had the highest upregulated expression in the MK compared to the FB and DK. Cytochrome P450 family proteins are necessary for many kinds of secondary metabolites biosynthesis. These genes were significantly expressed in the MK of *H. erinaceus*, indicating that the MK might produce more secondary compounds. MnP is known as an important biotechnological enzyme and has been isolated from many species of white-rot fungi^27^. High expression of MnP in the MK indicates its strong degradation capability. In addition, glycoside hydrolase and glutathione S-transferase were highly expressed in the DK. In the FB stage, the genes encoding 2-deoxyglucose-6-phosphate phosphatase, high mobility group B protein, oxalate decarboxylase, extracellular metalloproteinase, zinc finger protein and other hypothetical proteins were highly expressed.

### Prediction of gene clusters involved in bioactive secondary metabolite biosynthesis of *H. erinaceus*

**Figure 2:**
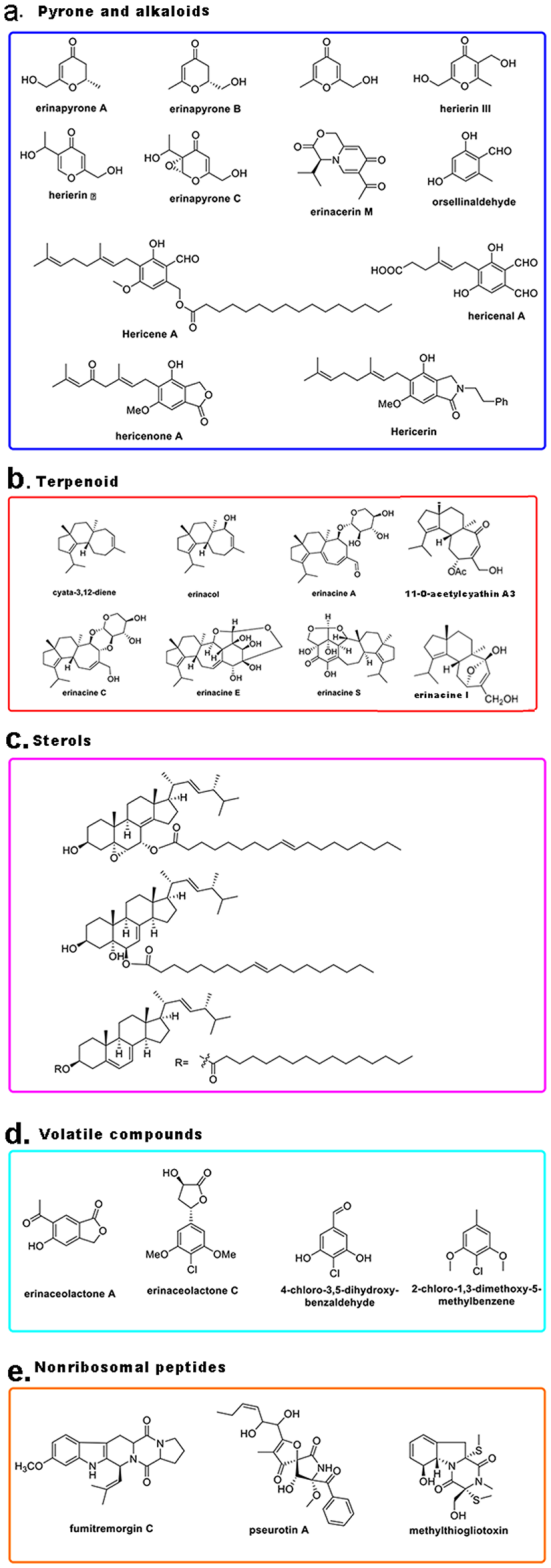
The typical bioactive secondary compounds isolated from *Hericium erinaceus*. (**a**) Pyrone and alkaloids: erinapyron A–C, herierin III; hericene A. (**b**) Terpenoid: erinacines A–E; cyatha-3,12-diene. (**c**) sterol compounds. (**d**) Volatile aromatic compounds: erinaceolactone A, C. (**e**) Nonribosomal peptides: fumitremorgin C and methylthiogliotoxin.

*H. erinaceus* FLM and FB contain a large number of structurally diverse bioactive secondary metabolites. These compounds mainly include pyrones, alkaloids, terpenoids, steroids, volatile aromatic compounds and nonribosomal peptides (NRPS) (Fig. 2). In particular, the production of the unique cyathane-type diterpenoids medicinal compounds (erinacines) by *H. erinaceus* indicated that this fungus probably had specific and functional diterpenoid synthases.

In fungi, genes involved in secondary metabolite biosynthesis are organized in discrete clusters around synthase genes^28^. However, the high degree of complexity and variability of fungal gene clusters and the fact that few Basidiomycota gene clusters have been fully characterized makes it difficult to identify biosynthetic gene clusters for this group of fungi. In our study, we predicted the genes and gene clusters involved in terpenoid and polyketide biosynthesis using a homology sequence search method (BLAST) and antiSMASH 3.0 software^29^. In brief, genes related to terpenoid backbone and sterol biosynthesis were identified using BLAST against the KEGG annotation (Tables 2–4). Geranylgeranyl pyrophosphate synthase (GGPPS) is usually contained in the diterpene biosynthesis gene clusters of fungi and bacteria; thus, the diterpene gene cluster was identified by manually scanning the genome sequence 20 kb upstream and downstream of predicted GGPPS sequences. The sesquiterpene synthases were identified based on multiple sequence alignments and phylogenetic analyses developed by the Schmidt-Dannert group^30, 31^. The terpenoid and PKS gene clusters were also predicted by antiSMASH 3.0 software.

**Table 2:**
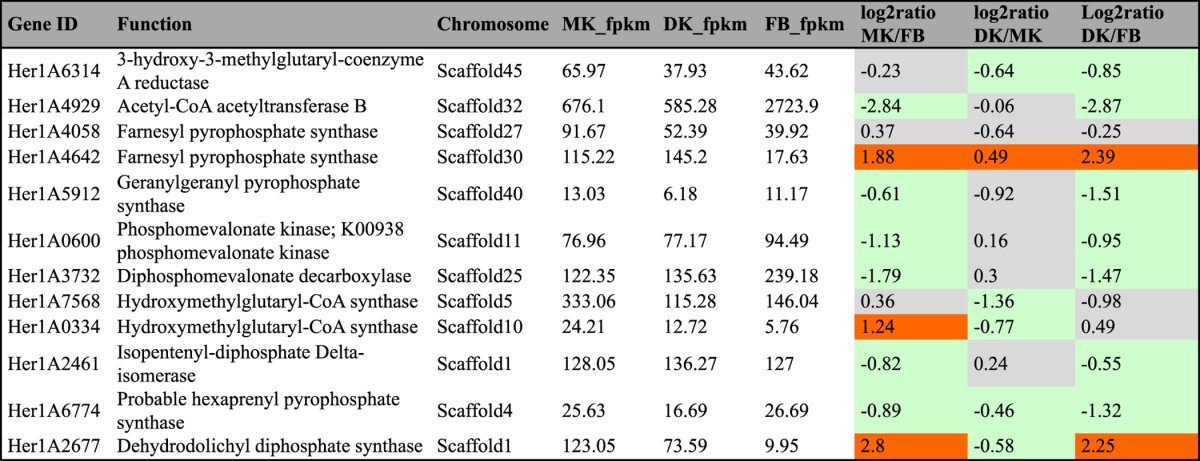
Genes and enzymes invovled in terpenoid backnone biosynthesis in KEGG pathway. #fpkm value is the mean expression value of three biological replicates. ^#^Abbreviation: MK: monokaryontic mycelium; DK: dikaryontic mycelium; FB: fruiting body. ^#^Orange color means genes significantly up-regulated; ^#^Green color means genes significantly down-regulated; ^#^Gray color means genes no significantly differential expression.

**Table 3:**
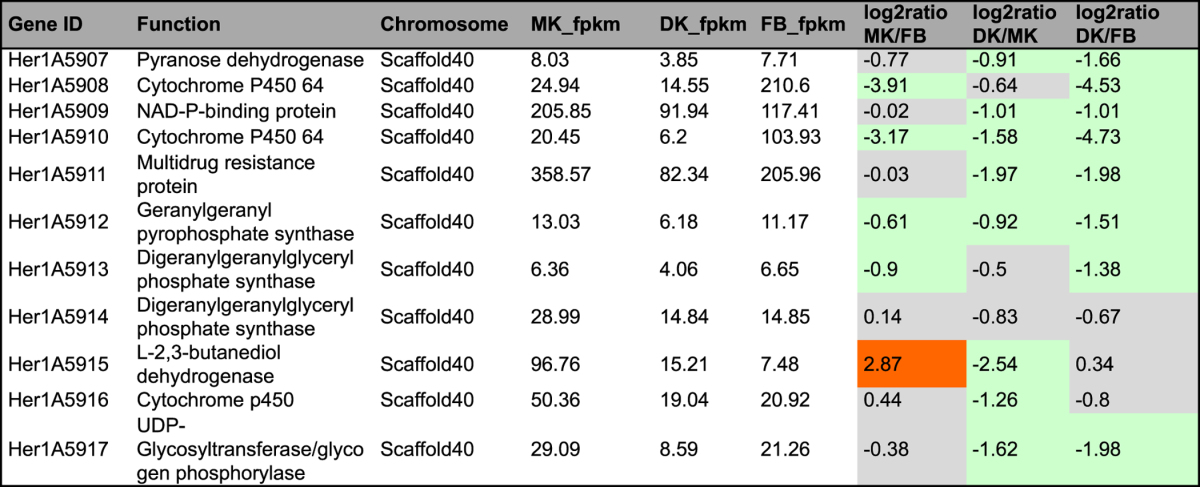
11 genes and enzymes involved in diterpenoid biosynthesis in *H. erinaceus*. ^#^fpkm value is the mean expression value of three biological replicates. ^#^Abbreviation: MK: monokaryontic mycelium; DK: dikaryontic mycelium; FB: fruiting body. ^#^Orange color means genes significantly up-regulated; ^#^Green color means genes significantly down-regulated; ^#^Gray color means genes no significantly differential expression.

**Table 4:**
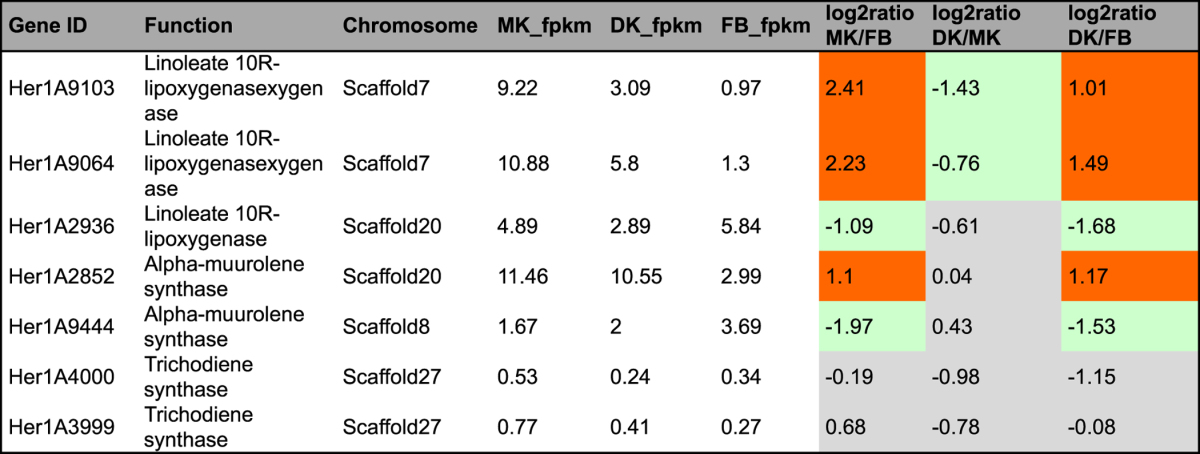
Seven genes and enzymes involved in sesquiterpenoid biosynthesis in *H. erinaceus*. ^#^fpkm value is the mean expression value of three biological replicates. ^#^Abbreviation: MK: monokaryontic mycelium; DK: dikaryontic mycelium; FB: fruiting body. ^#^Orange color means genes significantly up-regulated; ^#^Green color means genes significantly down-regulated; ^#^Gray color means genes no significantly differential expression.

### Terpenoid backbone synthesis genes

**Figure 3:**
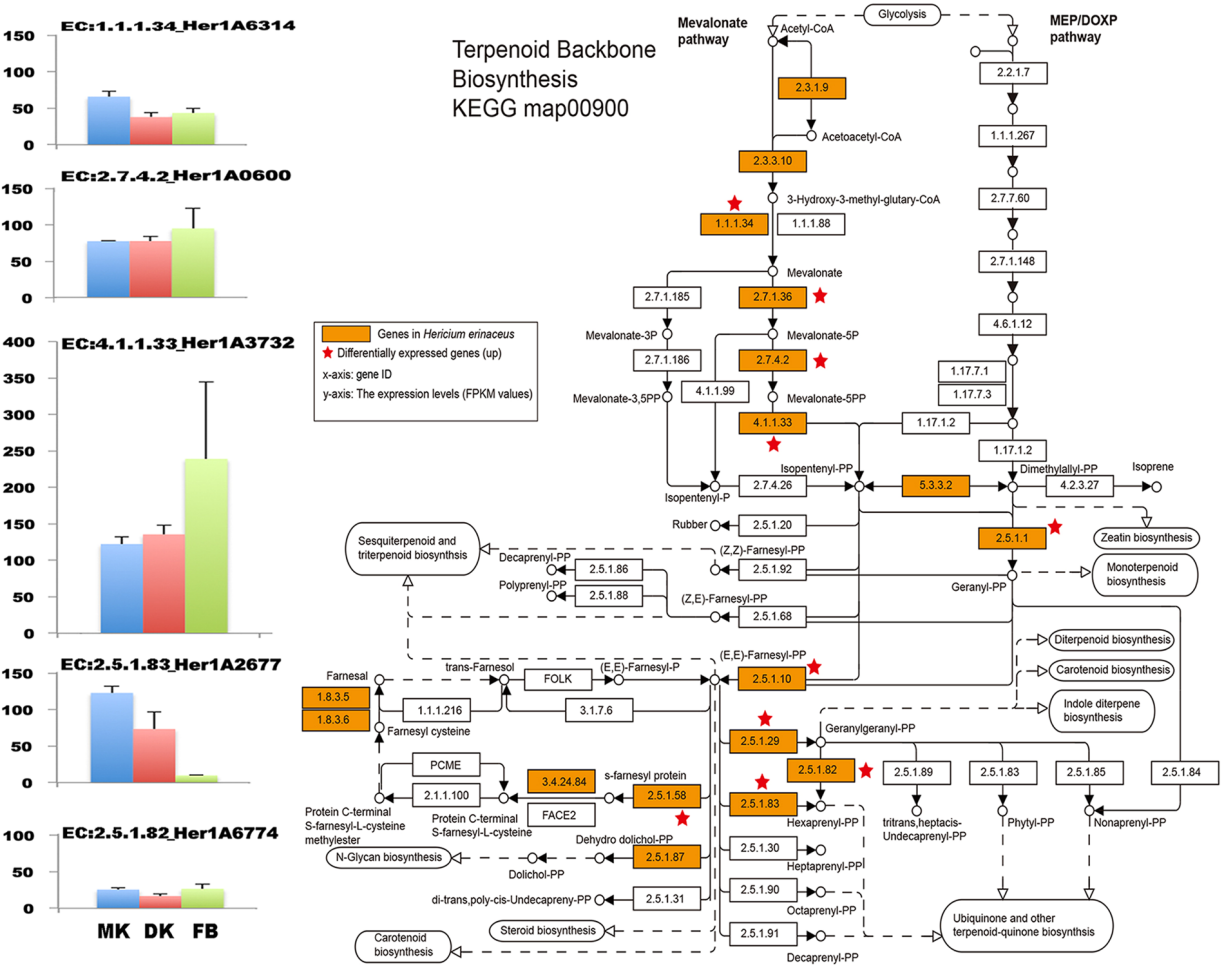
KEGG mapping of terpenoid backbone biosynthesis pathway identified in *Hericium erinaceus* and the putative gene expression level on different tissues. (Right) KEGG map 00900^23^. Red stars indicate the hits of differentially expressed genes in this map. (Upper Left) The expression levels (fpkm values) of mapped genes (EC 1.1.1.34, EC 2.5.1.10, EC 2.5.1.1, EC 2.7.4.2, EC 4.1.1.33, EC 2.5.1.82, EC 2.5.1.83, EC 1.8.3.5) of in different tissues. Abbreviation: MK, monokaryotic mycelium; DK, dikaryotic mycelium; FB, fruiting body.

We collected core enzymes involved in terpenoid backbone biosynthesis in KEGG pathway data and found 12 genes responsible for terpenoid backbone synthesis, including farnesyl pyrophosphate synthase and geranylgeranyl pyrophosphate synthase, the two key enzymes for biosynthesis of the common precursor GGPP for diterpenoid synthesis. The genes encoding farnesyl pyrophosphate synthase (HerlA4642) and dehydrodolichyl diphosphate synthase (Her1A2677) were upregulated in the FLM with a more than 4.0-fold change compared to that in the FB. The acetyl-CoA acetyltransferase (HerlA4929) had increased transcription in the FB stage, with an approximately 7-fold change compared to that in the FLM (Table 2, Fig. 3). Taken together, genes encoding tepenoid synthase were usually upregulated in the FLM, indicating that tepenoid compounds are probably more abundant in the FLM than in the FB of *H. erinaceus*.

### Diterpenoid pathway genes search and analysis

Diterpenoids are classically defined by 20-carbon isoprenoids synthesized from the common precursor geranylgeranyldiphosphate (GGPP) by the mevalonate (MEP) pathway in plants and fungi. Diterpene synthases (DiTPSs), diterpene cyclases and cytochrome P450-dependent mono-oxygenases (P450s) are the major enzyme modules to generate diterpenoid diversity^32^. In plants, the well-known diverse diterpene synthases include taxadiene synthase (TS), ent-copalyl diphosphate synthase (ent-CPS), and ent-kaurene synthase (ent-KS)^33, 34^. However, compared with the plant diterpene synthases and diterpene cyclases, these enzymes from basidiomycetes fungi and bacteria are seldom studied.

To search for genes involved in diterpenoid biosynthesis in *H. erinaceus*, we took the fungal diterpene synthase genes sequences cited in Fischer *et al*.^35^ and the sequence of a GGPPS (Pl-ggs) from the basidiomycete *Clitopilus passeckerianus* ^36^ as queries to perform homology searches against the amino acid sequence of the *H. erinaceus* genome. A gene encoding geranylgeranyl pyrophosphate synthase (GGPPS) (Her1A5912) with high homology (35%) was identified and another 10 genes around the core gene were identified by manually scanning the genome sequence 20 kb upstream and downstream of the predicted GGPPS sequences (Table 3). The predicted gene cluster for diterpene biosynthesis is located in scaffold 40 and included a GGPPS, three cytochrome P450s, two digeranylgeranylglyceryl phosphate synthases, a UDP-glycosyltransferase/glycogen phosphorylase, an L-2,3-butanediol dehydrogenase, a pyranose dehydrogenase and a multidrug resistance protein (Table 3). The two cytochrome P450s (Her1A5908 and Her1A5910) were upregulated in the FB compared to the FLM in *H. erinaceus*, with a more than 20-fold change (Table 3). The gene encoding the L-2,3-butanediol dehydrogenase (Her1A5915) had upregulated expression in MK compared to FB and DK. In most previous studies, diterpenoids compounds were mainly isolated from the FLM of *H. erinaceus* ^37^. Sequencing analysis of the *H. erinaceus* genome showed that genes involved in diterpenoid synthesis had diverse expression in the FLM and FB.

### Genes for sesquiterpene biosynthesis

**Figure 4:**
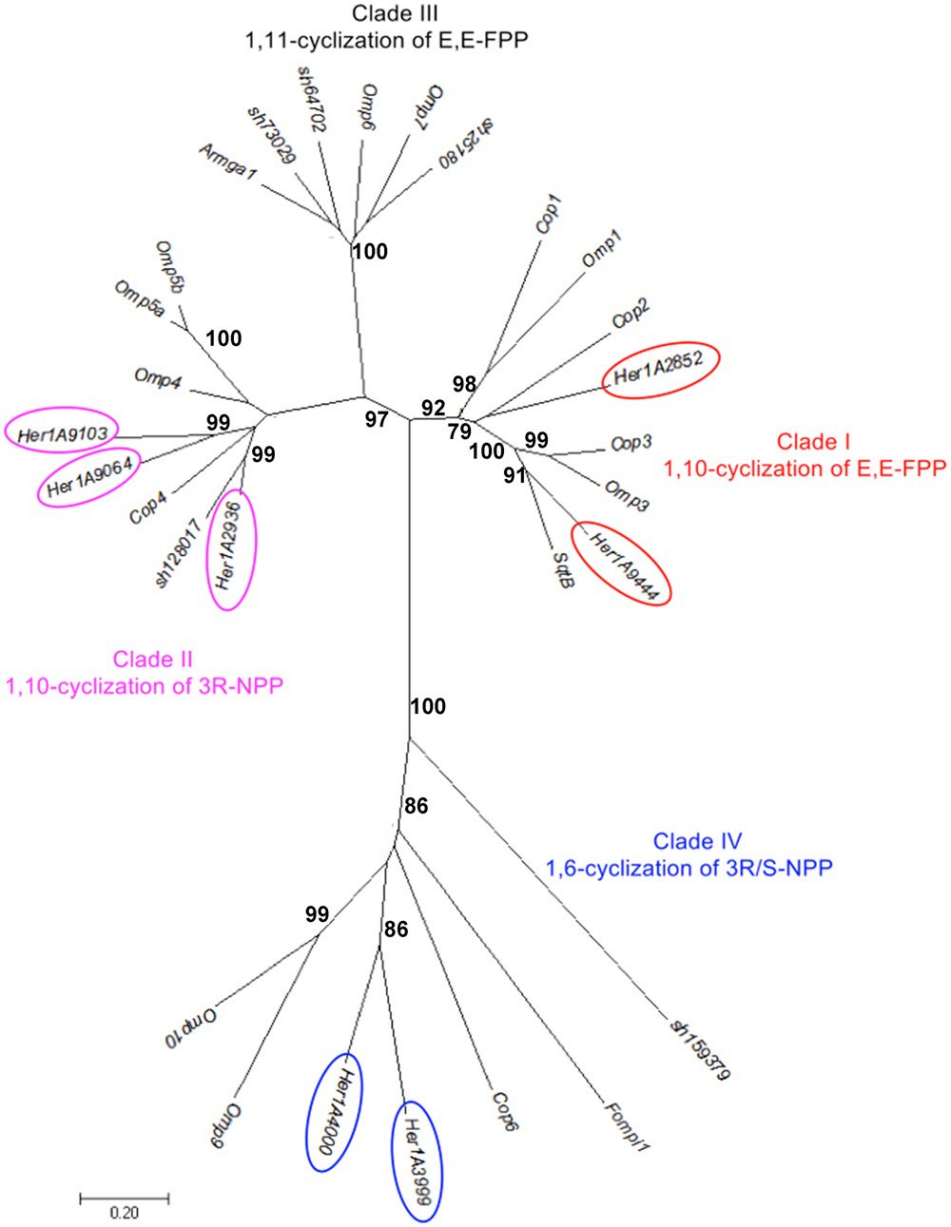
Unrooted Neighbor-Joining phylogram of sesquiterpene synthase (STS) of *Hericium erinaceus* were constructed based homologous protein sequences. STS from *Coprinopsis cinereus* (Cop), *Omphalotus olearius* (Omp), *Fomitopsis pinicola* (Fompi1),﻿ *Stereum hirsutum* (Sh) and the single STS described from *Armillaria gallica* (Armga1) and *Boreostereum vibrans* (SqtB) are labeled. Detail information of the sequences used in phylogram can be found in Supplement Table S4.

Sesquiterpene compounds have been reported from different Basidiomycota species as mainly bioactive secondary metabolites^38^. Sesquiterpene scaffolds are synthesized from the prenyl compound farnesyl diphosphate (FPP) by a class of enzymes known as sesquiterpene synthases (STS)^31^. Seven homologous sequences with considerable similarity (P < 10^−5^ e-value) to the known, biochemically characterized sesquiterpene synthases were identified in the genome of *H. erinaceus* (Table 4). The phylogenetic analysis showed that these genes consist of 4 clades (clade I-IV) and genes isolated from *H. erinaceus* belonged to 3 clades (clade I, II, IV), representing 3 functional groups (Fig. 4, Supplementary Table S4)^31^. The seven STS genes of *H. erinaceus* include three genes encoding linoleate 10R-lipoxygenase (Her1A9103, Her1A9064, Her1A2936 in clade II), two genes encoding alpha-muurolene synthase (Her1A2852, Her1A9444 in clade I) and two genes encoding trichodiene synthase (Her1A4000 and Her1A3999 in clade IV). Among them, two genes encoding linoleate10R-lipoxygenase (clade II) and a gene encoding alpha-muurolene synthase (clade I) were mainly upregulated in the FLM compared to the FB (Table 4). Although few studies have reported sesquiterpene compounds from *H. erinaceus*, genes encoding linoleate 10R-lipoxygenase and alpha-muurolene synthase for sesquiterpene biosynthesis displayed relatively high expression in the FLM compared to the FB of *H. erinaceus*. However, the expression of trichodiene synthase and other sesquiterpene cyclases has not previously been detected in the FLM or FB of *H. erinaceus*. In addition, the predicted genes involved in sesquiterpene biosynthesis do not cluster in the *H. erinaceus* genome. Seven genes encoding STS were located on four different scaffolds of *H. erinaceus* genome (scaffolds 7, 8, 20 and 27).

**Figure 5:**
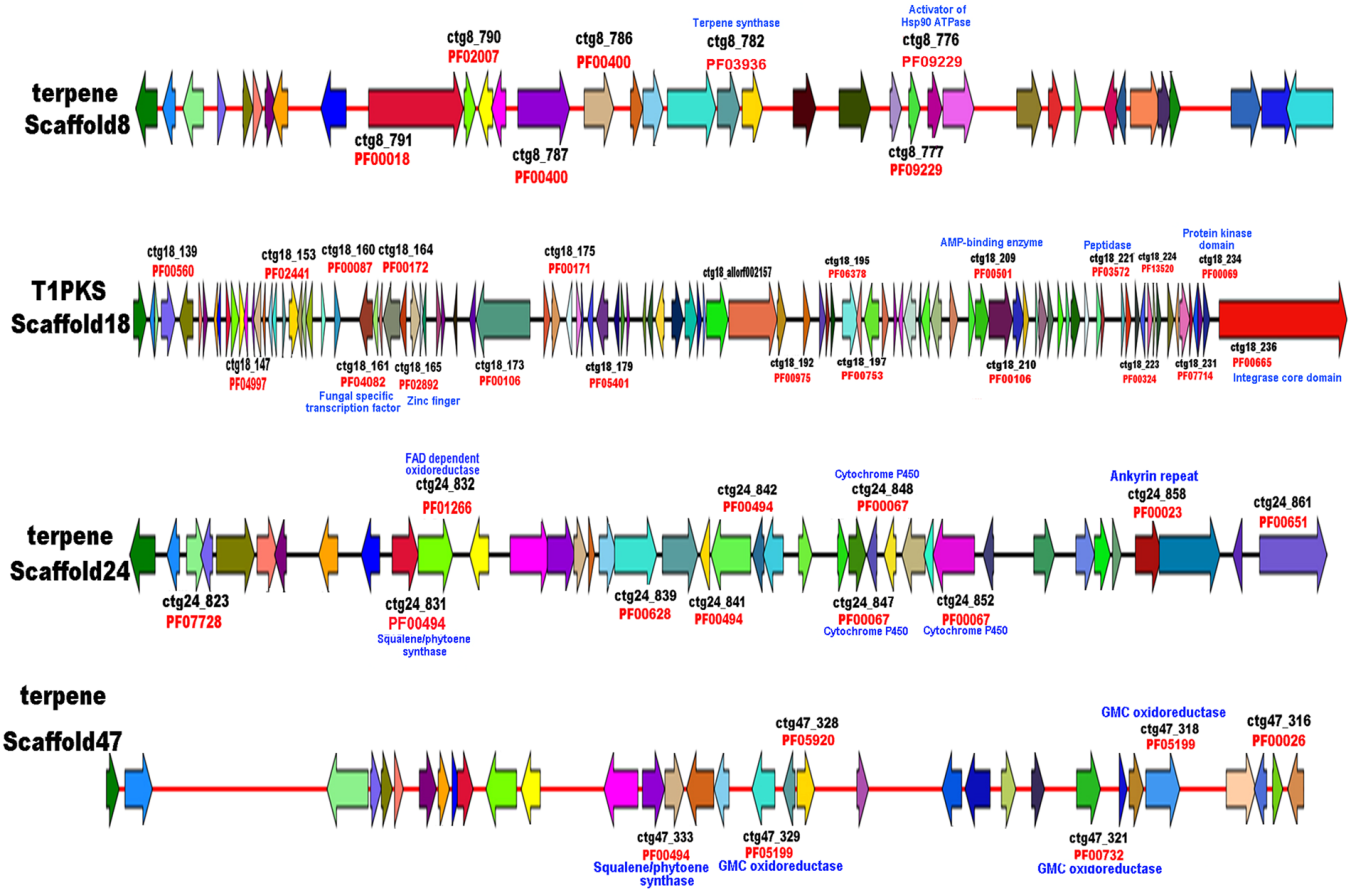
Identification of the four putative gene clusters for terpene and polyketides (PKS) in *Hericium erinaceus* genome by antiSMASH software. ctg ID (black font) in the map represents coding sequence (cds) place in corresponding scaffold. Genes with Pfam accession number (red color) and functional annotation (blue color) were marked in the map. The detail annotation of genes in each cluster was listed in Supplementary Table S5.

We also identified the gene clusters involved in terpenoid biosynthesis using the antiSMASH software. Three gene clusters were predicted to be involved in terpenoid biosynthesis. These clusters were located on three scaffolds (scaffold 8, 24 and 47) within well-defined cluster and each cluster spanned 20 kb of *H. erinaceus* genome (Fig. 5). Genes with Pfam functional annotation are marked in Fig. 5 and Supplementary Table S5. The gene cluster for terpene biosynthesis in scaffold 8 included a gene encoding terpene synthase, an activator of Hsp90 ATPase and other genes with unknown function. The gene cluster in scaffold 24 contained the core gene encoding squalene/phytoene synthase, 2 FAD dependent oxidoreductases, 3 CYP450 genes and other genes with unknown function. The gene cluster in scaffold 47 included 6 known functional genes (e.g., a squalene/phytoene synthase, three GMC oxidoreductases, a eukaryotic aspartyl protease and a homeobox KN domain protein) (Supplementary Table S5).

### Polyketide biosynthesis

In addition to terpenoid compounds, polyketides are another major group of secondary metabolites isolated from *H. erinaceus*, including hericenones, hericerins and hericenes^1^. AntiSMASH predicted one cluster responsible for PKS synthesis located on a single scaffold 18 and in a well-defined cluster spanning approximatly 7.2 kb (Fig. 5, Table S5). The gene cluster include core genes encoding polyketide synthase (Her1A2028), aldehyde dehydrogenase family genes and a fungus-specific transcription factor domain, fungal Zn(2)-Cys(6) binuclear cluster domain gene. Polyketide synthase is upregulated in the FB compared to FLM (Table 5); these results are consistent with the hericenone compounds being easily isolated from the FB of *H. erinaceus* ^39^. In addition, a gene encoding midasin showed high expression in the FB, suggesting a role in basidiomatal formation. Furthermore, midasin was also reported to be involved in female gametogenesis of *Arabidopsis thaliana* ^40^.

**Table 5:**
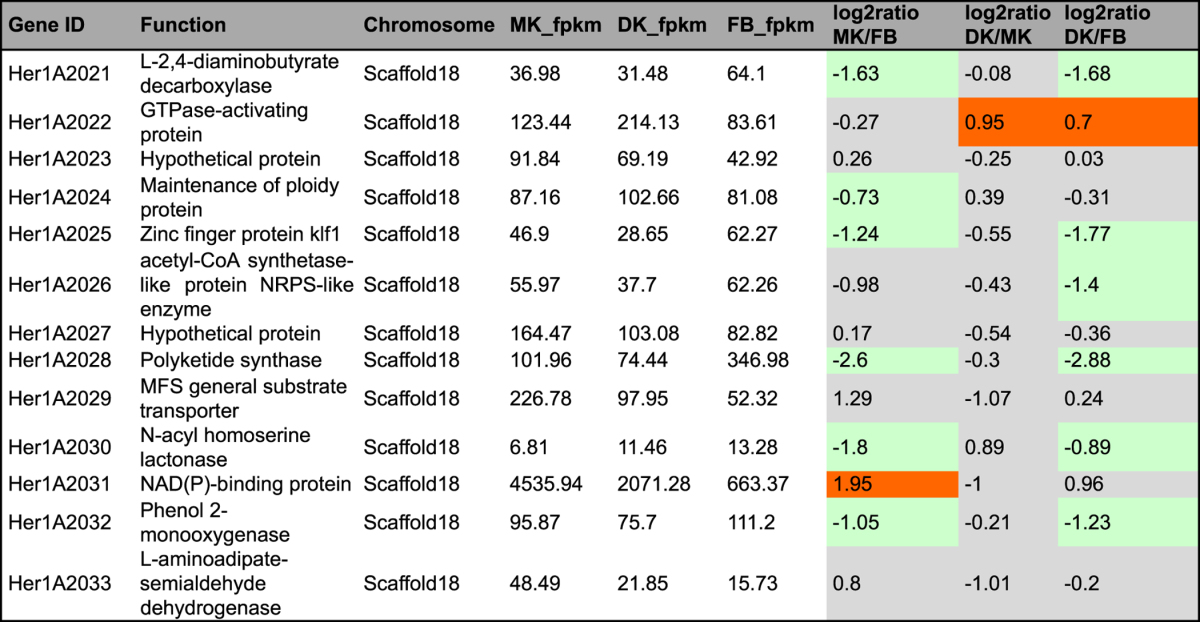
Gene cluster invovled in polyketides biosynthesis of *H. erinaceus*. ^#^fpkm value is the mean expression value of three biological replicates. Abbreviation: MK: monokaryontic mycelium; DK: dikaryontic mycelium; FB: fruiting body. ^#^Orange color means genes significantly up-regulated; ^#^Green color means genes significantly down-regulated; ^#^Gray color means genes no significantly differential expression.

### Sterols

In recent years, various sterols have been identified from *H. erinaceus*. For example, Avtonomova *et al*.^41^ detected the presence of ergosterol in *H. erinaceus* mycelia, although the amounts of such bioactive sterols were fairly low. Li *et al*.^16^ isolated a series of sterol compounds with long chain fatty acids from the FB of *H. erinaceus* and some of these sterols have good cytotoxic activity. We identified 18 genes involved into sterol biosynthesis from the *H. erinaceus* genome, including sterol O-acyltransferase, lanosterol synthase, C-8 sterol isomerase, C-5 sterol desaturase, and C-22 sterol desaturase (Table 6). C-8 sterol isomerase is a key enzyme responsible for transformation from fecosterol to episterol, and C-5 sterol desaturase is an enzyme that catalyzes the dehydrogenation of a C-5 (6) bond in a sterol intermediate compound in the biosynthesis of major sterols.

**Table 6:**
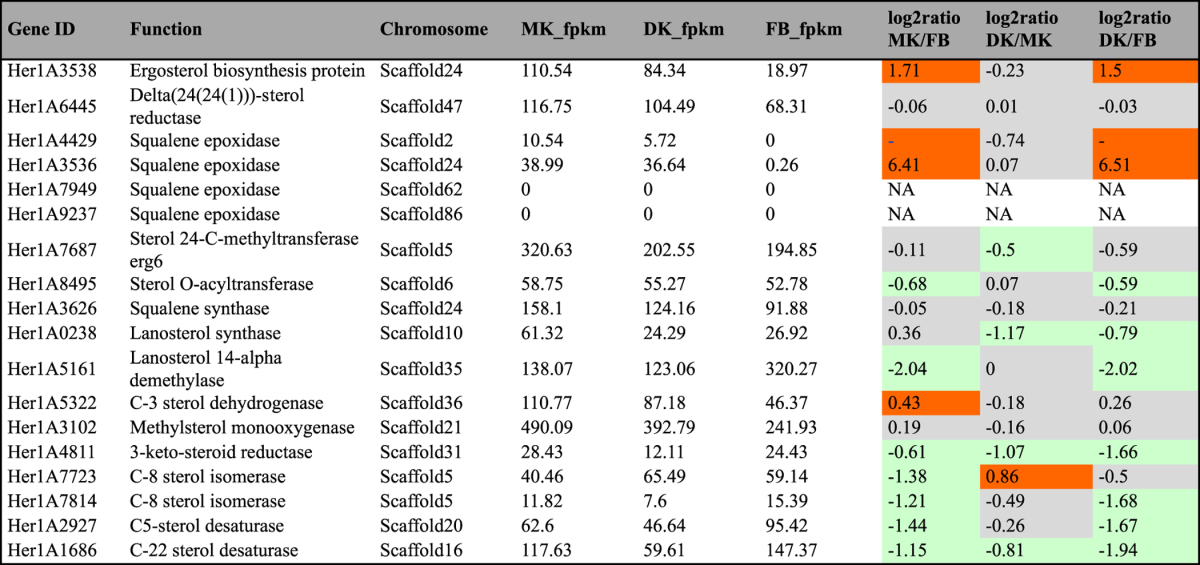
Genes and enzymes invovled in sterol biosynthesis in KEGG pathway. ^#^fpkm value is the mean expression value of three biological replicates. Abbreviation: MK: monokaryontic mycelium; DK: dikaryontic mycelium; FB: fruiting body. ^#^Orange color means genes significantly up-regulated; ^#^Green color means genes significantly down-regulated; ^#^Gray color means genes no significantly differential expression.

A total of 4 genes encoding squalene epoxidase were identified in *H. erinaceus* but only 2 genes were detected with low expression levels in the FLM (FPKM < 40) but silent in the FB. Squalene epoxidase is a key regulatory enzyme in triterpenoid saponin biosynthesis and catalyzes the epoxidation of squalene to form oxidosqualene. These results help explain why few triterpenoid compounds were isolated from this mushroom compared to other medicinal mushrooms (e.g., *Ganodema lucidum* and *Antrodia cinnamomea*).

### Cytochrome P450s

Cytochrome P450s (CYPs) play essential roles in biosynthesis of fungal secondary metabolites. By homology BLAST in the Fungal Cytochrome P450 Database (p450.riceblast.snu.ac.kr/index.php? a = view), we obtained 137 gene IDs. They were classified into at least 4 subfamilies, including CYP2, CYP3/CYP5/CYP6/CYP9, CYP4/CYP19/CYP26 and CYP11/CYP12/CYP24/CYP27 (Supplementary Table S6). Coupled with KEGG pathway analysis, a total of 137 CYP450 genes mainly participating in secondary metabolite biosynthesis, amino acid transport, energy production and inorganic ion transport were identified. Hierarchical clustering showed that 137 CYP450 genes were grouped into 7 clades based on their expression profiles (Supplementary Fig. S5). Genes in clade 2 are highly expressed across three different tissues (MK, DK and FB) of *H. erinaceus*, including psi-producing oxygenase, lanosterol 14-alpha demethylase, apoptosis inducing factor, putative aldehyde dehydrogenase-like protein, endonuclease, and benzoate 4-monooxygenase etc. (Supplementary Fig. S5). Psi-factor-producing oxygenases (Ppos) are fusion proteins consisting of a peroxidase-like functionality in the N-terminus and a P450 fold in the C-terminal part of the polypeptide chain^42^.

Lanosterol 14-alpha demethylase (CYP51) is responsible for an essential step in the biosynthesis of sterols by catalyzing the demethylation of lanosterol to produce an important precursor that is eventually converted into ergosterol^43^. Genes in clade 3 and clade 5 are highly expressed in the FLM, including three genes encoding ent-kaurene oxidase (Her1A5449, Her1A5450 and Her1A8895) for diterpenoid biosynthesis. We identified 28 monooxygenase candidates, including *O*-methylsterigmatocystin oxidoreductase, lanosterol 14-alpha demethylase and fumitremorgin C synthase etc. These genes had diverse expression in different *H. erinaceus* tissues but were usually highly expressed in the FLM. Fumitremorgin C synthase was reported to catalyze the conversion of tryprostatin A to fumitremorgin C and is probably involved in alkaloid biosynthesis^13^. Moreover, two fumitremorgin C synthase genes are upregulated in the mycelia stage with more than 200-folds change compared to the FB (Her1A1055).

### Comparative analysis of Basidiomycota genomes uncovers unique biosynthetic genes of secondary metabolites in *H. erinaceus*

**Figure 6:**
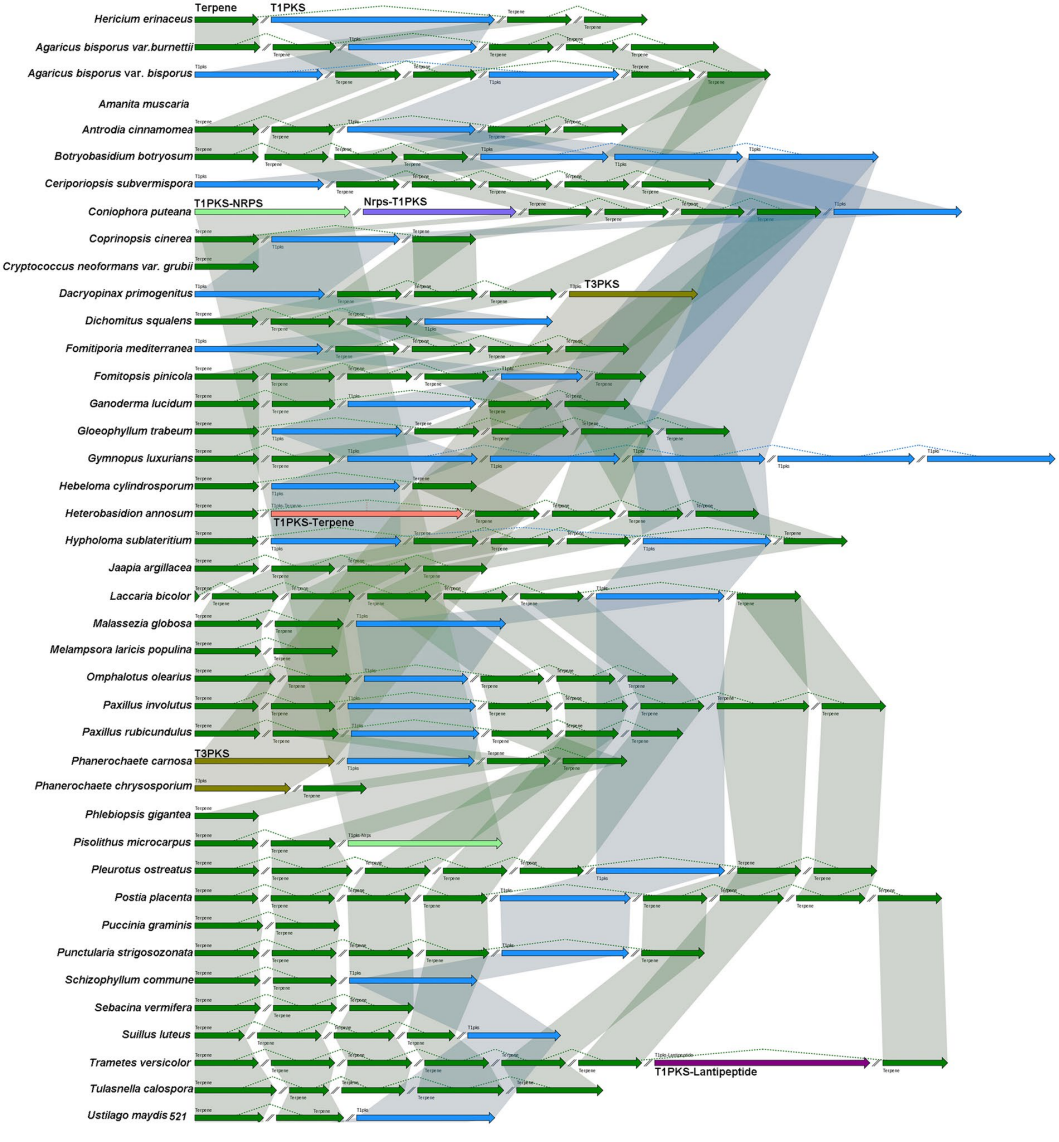
An ideogram showing the putative gene clusters involved in secondary metabolic biosynthesis (terpenoid and polyketides) in 42 basidiomycota fungi. The gene clusters of each fungal species were predicted by antiSMASH software and then the syntenic picture was drew using the SVG tool based on the these gene clusters. The square boxes refer to the entire gene cluster involved in secondary metabolism in each fungal species: terpenoid gene cluster (green color); type I PKS gene cluster (blue color); NRPS-type I PKS gene cluster (purple color); TypeIII PKS (yellow brown color) and type I PKS-terpene gene cluster (pale red color). The shaded regions refer to presumably syntenic or homologous gene clusters between fungal species and the same type of gene clusters were connected by thin lines. The symbol “//” was used to separate the gene cluster in different scarffold. # no gene cluster for secondary metaboites biosynthesis was predicted in the *Piriformaspora indica* and it is not be shown in the figure.

To better understand the natural product biosynthesis in Basidiomycota, we conducted a comparative genomic analysis of terpenoid and polyketides biosynthesis including another 41 Basidiomycota. Genome information of the 42 fungi is listed in Supplementary Table S7. The genome sequence of *H. erinaceus* revealed 4 large genes clusters located on four different scaffolds for terpenoid and polyketide biosynthesis (Fig. 5), including a type1 PKS gene cluster and three terpenoid gene clusters. Comparative genomic analysis revealed that no gene clusters related to terpenoid or polyketides biosynthesis was found in the genome of the endophytic fungus *Piriformospora indica* or the poisonous fungus *Amanita muscaria* (Fig. 6). The medicinal fungi, *H*. *erinaceus*, *Antrodia cinnamomea* and *Ganoderma lucidum* have 3-4 gene clusters for terpenoid and a single gene cluster for polyketides biosynthesis. The larger number of terpenoid and type I PKS gene clusters in the *H. erinaceus* genome supports polyketide and terpenoid compounds as major classes of secondary metabolites in this fungus. Comparative genome analysis of secondary metabolites biosynthesis also provides important information for effective utilization of functional genes for natural product biosynthesis using medicinal fungi in the future.

### Genes putatively involved in sexual reproduction in the *H. erinaceus* genome

The sexual reproduction of *H. erinaceus* is typical of a heterothallic basidomycete. Thus, mating and subsequent FB development occur only after fusion of mycelial structures of opposite mating types^44^. Molecular mechanisms of fruiting body development in Basidiomycota are poorly known. To search for genes involved in *H. erinaceus* sexual reproduction, we collected *Aspergillus nidulans* sexual reproduction-related protein sequences from the NCBI database as reference^45^. By BLASTP analysis of this query dataset against the *H. erinaceus* genome annotation file, we identified 41 sequences putatively encoding genes involved in the sexual reproduction of *H. erinaceus* (Supplementary Table S8). In the monokaryotic strain Her1 of *H. erinaceus* genome, only mating type gene MAT1-2-1 (Her1A1771), encoding a high mobility group DNA binding protein was identified. This gene showed significantly upregulated expression in the MK and DK stage compared to the FB stage but was down regulated in the DK compared to the MK (Supplementary Table S8).

Genes encoding G protein receptors, pheromones, and transcription factors have been confirmed to be involved in complex developmental programs^46^. In the *H. erinaceus* genome, a putative gene encoding pheromone maturation dipeptidyl aminopeptidase (Her1A8352) was identified and had increased expression in the fruiting body stage. In addition, four genes related to mating signal transduction, including pheromone a factor receptor PreA/Ste6 (Her1A4437), mitogen activated protein kinase Ste11 (Her1A5786), mitogen activated protein kinase kinase (Her1A2154) and sexual development transcription factor Ste12 (Her1A4410) were also found in the *H. erinaceus* genome. Among them, two mitogen activated protein kinase genes were both upregulated in the FB of *H. erinaceus* (Supplementary Table S8), implying an important role in the sexual reproduction of fungi.

Meiosis is a key feature of eukaryotic sexual reproduction. We also surveyed the *H. erinaceus* genome for the proteins that constitute the conserved meiotic machinery in eukaryotic organisms (particularly *Saccharomyces cerevisiae* and *S. pombe*). Twenty-three genes homologous to *S. cerevisiae* or *S. pombe* meiotic genes were found in the *H. erinaceus* genome (Supplementary Table S8), including genes encoding anaphase-promoting complex (APC) components (Her1A5674, Her1A2492, Her1A2965, Her1A4081), B-type cyclin proteins, sporulation-regulated septins, spindle pole body components and chromosome segregation proteins. These genes were highly expressed in the FB compared to the FLM (Supplementary Table S8). B-type cyclin is considered a positive regulatory subunit of Cdc28/Cdc2, while *CDC28*-encoded protein kinases are necessary for mitosis events in *S. cerevisiae* ^47^. In addition, two genes homologous to core meiotic genes in budding yeast (an RNA binding protein required for meiotic recombination, Her1A2571, and a protein required for synaptonemal complex formation, Her1A3304) were also found in the *H. erinaceus* genome and are upregulated in the fruiting body (Supplementary Table S8).

## Discussion

In this study, we carried out the *de novo* genome sequencing, assembly, and annotation of *H. erinaceus*, a medicinal and wood rot fungus in the family Hericeaceae of Basiciomycota. Genome content and structure of *H. erinaceus* are similar to other several medicinal wood-fungi, such as *A. cinnamommea* and *G. lucidum*. It is also the second genus to have a known genomic sequence in the Hericeaceae, after *Dentipellicula*. Our study provides an important dataset for fungal phylogenetic evolution and reconstruction at the genome level.

We conducted a prediction of gene clusters involved in secondary metabolism and identified many genes encoding protein candidates for the terpenoid, polyketide and sterol biosynthesis. These included geranylgeranyl pyrophosphate synthase (Her1A5912), responsible for the biosynthesis of common precursor GGPP for diterpene compounds production, and sesquiterpene synthases (e.g., linoleate 10R-lipoxygenase and alpha-muurolene synthase) (Table 4). Some genes displayed different expression levels in different tissues of *H. erinaceus*. For example, genes responsible for terpenoid biosynthesis were usually highly expressed in the FLM while PKS genes involved in polyketides biosynthesis were mainly upregulated in the FB stage. Tissue-specific transcript profiling across three different tissues of *H. erinaceus* revealed that the gene expression dynamics are probably associated with bioactive compounds biosynthesis by tissue-specific pathways.

Few studies have reported that sesquiterpene compounds were isolated from *H. erinaceus*. In this study, 7 STS involved in sesquiterpene biosynthesis were identified from the *H. erinaceus* genome. Of these, five genes are differentially expressed in the three tissues of. *H. erinaceus*. However, these genes usually have low expression with fpkm values less than 15.0. These results indicated that the fungus has potential to produce low quantities of sequiterpenes in our culture condition.

Cyathane-type diterpenes, which have a 5-6-7 tricyclic skeleton, are considered to be unique and highly bioactive secondary metabolites in *H. erinaceus*. We identified a gene cluster for diterpene biosynthesis in the genome of *H. erinaceus*. Based on genome sequence analysis, we discovered and characterized a diterpenes cyclases for the biosynthesis of erinacines for the first time in this family of fungi^48^.

The polyketides of microorganisms and plants comprise a large and structurally diverse family of bioactive natural products, and they are indispensable for drug discovery^49^. For example, polyketide drugs include antibiotics, antifungals, immunosuppressants and anticancer agents. It is reported that structurally diverse polyketides compounds are made by type I modular polyketide synthases (PKSs) from simple metabolites such as propionyl-CoA, malonyl-CoA and methylmalonyl-CoA^50^. There are three types of PKSs (types I, II and III) and type I PKSs are typically found in fungi^51^. We also found a type I polyketide synthase in a well-defined gene cluster that displayed significantly upregulated expression in the FB of *H. erinaceus*. In addition, a large number of CYP450 genes were identified in the *H. erinaceus* genome. These genes played an essential role in the biosynthesis of terpenoids, sterols and nonribosomal peptides.

In the present study, we performed gene cluster prediction for secondary metabolite biosynthesis using homology sequence searches (BLAST) and the antiSMASH software. The results for terpenoid synthesis gene clusters were not identical using the two methods (Table 2, Fig. 5). From the literature, antiSMASH has been trained for the identification of specific natural products gene clusters (e.g., PKS and NRPS) in certain groups of organisms (e.g., Ascomycota, bacteria), but it is not suitable for thoroughly at identifying terpenoid clusters in Basidiomycota^52^. Thus, in our study, the genes related to terpenoid backbone, diterpenoid, sesquiterpene and sterol biosynthesis were also identified based on sequence homology searches in addition to antiSMASH predictions. Overall, our study provides a comprehensive collection of key enzymes involved in production of the main bioactive metabolites of *H. erinaceus*. These datasets will be valuable for the biotechnology industry in producing and packaging these metabolites for commercial applications from mushrooms in the future and will improve our knowledge of *H. erinaceus* biology.

## Materials and Methods

### Origins of strains and culture conditions

The haploid monokaryotic strain *H. erinaceus* Her1 (deposited in Mycolab of Institute of Medicinal Plant Development, Chinese Academy of Medical Sciences) was grown on potato dextrose agar (PDA) at room temperature for 2–3 weeks in the dark (Supplementary Fig. S1). The fruiting bodies were obtained directly from commercial production. Internal transcribed spacer (ITS) was sequenced to confirm that these strains were the same species. The mycelia layer at the top of the culture was scraped off the petri dish, frozen in liquid nitrogen and ground to a fine powder for genome sequencing. Total genomic DNA was extracted using the Omega E.Z.N.A. fungal DNA midi kit (Omega, USA) from roughly 0.5 g of crushed tissue.

### Genome sequencing assembly

*De novo* genome sequencing, assembly and initial annotation were carried out at Beijing Novogene Bioinformatics Technology Co., Ltd. (Beijing, China). The genome of *H. erinaceus* was sequenced with MPS (massively parallel sequencing) Illumina technology. A paired-end library with an insert size of 500 bp was sequenced using an Illumina MiSeq with a PE300 strategy and the two mate-pair libraries with an insert sizes of 2 kb and 5 kb were sequenced using an Illumina HiSeq. 2500 with a PE125 strategy. Raw sequencing data was suffered from quality-filtered by removing the Illumina PCR adapter reads and low-quality reads, and 3,862 Mb clean data were assembled into genomic sequences using the software SOAPdenovo (http://soap.genomics.org.cn/soapdenovo.html). Gene prediction was performed by Augustus software (http://bioinf.uni-greifswald.de/augustus/). Genome annotation was performed with BLAST against the following databases: NR, KEGG, KOG, GO and Swiss-Prot.

### RNA-Seq sequencing, annotation and expression analysis

RNA was extracted from the monokaryotic, dikaryotic mycelium and the fruiting body using the RNAeasy Plant Mini Kit (Qiagen) according to the manufacturer’s protocols, respectively. Each sample had three replicates. RNA degradation and contamination were monitored on 1% agarose gels. RNA purity was checked using the NanoPhotometes spectrophotometer (IMPLEN, CA, USA), concentration was measured using QubiRNA Assay Kit in Qubit® 2.0 Fluorometer (Life Technologies, CA, USA) and integrity was assessed using the RNA Nano 6000 Assay Kit of the Bioanalyzer 2100 system (Agilent Technologies, CA, USA). A total amount of 3 μg RNA per sample was used as input material for RNA sample preparation. Sequencing libraries were generated using NEBNext® UltraTM RNA Library Prep Kit for Illumina® (NEB, USA) following manufacturer’s recommendations and index codes were added to attribute sequences to each sample. All nine RNA samples were subjected to RNA-Seq on the Illumina HiSeq. 2000 platform (Illumina, San Diego, CA, USA). Raw data (raw reads) in fastq format were firstly processed through in-house Perl scripts. Reads containing adapter, reads containing poly-N and low-quality reads were removed from the raw data and all the downstream analyses were done based on clean data with high quality. The paired-end clean reads were aligned to the *H. erinaceus* genome using TopHat v2.0.12^53^. HTSeq v0.6.1 software was used to count the read numbers mapped to each gene^54^. The FPKM value was used to calculate gene expression, and the upper-quartile algorithm was used to correct the gene expression.

Gene differential expression analysis was performed using the DESeq R package (1.18.0) using a corrected p-value^55^. Genes with an adjusted *P*-value < 0.05 found by DESeq were assigned as differentially expressed. Hierarchical clustering of gene expression was conducted using Genesis 1.7.7^56^.

### Gene cluster prediction related to bioactive secondary metabolites

For the gene cluster prediction for diterpene biosynthesis, we used the sequence of a GGPPS from the basidiomycete *Clitopilus passeckerianus* (*Pl-ggs*)^36^ as query to perform homology searches of the *H. erinaceus* assembly. After that, we manually scanned the genome sequence 20 kb upstream and downstream of predicted GGPPS sequences. The sesquiterpene synthases were identified based on multiple sequence alignments and the phylogenetic analysis developed by the Schmidt-Dannert group^31^. Sequences of sesquiterpene synthases from *Coprinopsis cinereus, Omphalotus olearius* and *Stereum hirsutum* were used to perform homology searches in the *H. erinaceus* genome sequence using BLAST software. Phylogenetic analysis of sesquiterpene synthases was conducted using MEGA version 7.0 software^57^ with the default parameters for the Neighbor-Joining method with a bootstrap test of phylogeny (1000 replicates). The PKS gene cluster was identified by antiSMASH 3.0.5 software. The result was manually scanned 20 kb up-/downstream to confirm the association with putative PKS. Comparative genomic analysis of putative gene clusters involved in secondary metabolic biosynthesis was performed using antiSMASH (3.0.5) software. In brief, tepenoid and PKS gene cluster was predicted for each fungal species in genome scale to get the placement information for each gene cluster in scaffold; then we figured out an ideogram using the Sharp Vector Graphics tool (SVG).

The sequences reported in this paper have been deposited at GenBank database (Genome project: PRJNA361338; Transcriptome project: PRJNA361340).

## Electronic supplementary material

Supplementary information

Table S1

Table S2

Table S3

Table S4

Table S5

Table S6

Table S7

Table S8
