# Supplementary material for: Genomic and transcriptomic analyses reveal differential regulation of diverse terpenoid and polyketides secondary metabolites in Hericium erinaceus
Source: Sci Rep. 2017 Aug 31;7:10151. doi: 10.1038/s41598-017-10376-0 (PMC5579188; doi:10.1038/s41598-017-10376-0)
Supplement: Supplementary file 1 — Supplementary information [file 41598_2017_10376_MOESM1_ESM.pdf]

Supplementary Information

Genomic and transcriptomic analyses reveal differential regulation of diverse terpenoid and polyketides secondary metabolites in *Hericium erinaceus*

Juan Chen[1]*, Xu Zeng [1], Yan Long Yang[2], Yong Mei Xing[1], Zhang Qi[1], Jia Mei Li[1], Ke Ma[2], Hong Wei Liu[2], Shun-Xing Guo[1]*

1. Key Laboratory of Bioactive Substances and Resource Utilization of Chinese Herbal Medicine, Ministry of Education, Institute of Medicinal Plant Development, Chinese Academy of Medical Sciences & Peking Union Medical College, Beijing, 100193, P. R. China

2. State Key Laboratory of Mycology, Institute of Microbiology, Chinese Academy of Sciences, Beijing, 100101, P. R. China

*Corresponding author: kibchenjuan@126.com; sxguo1986@163.com;

Phone: +86 -10-57833240

Fax: +86-10-57833231

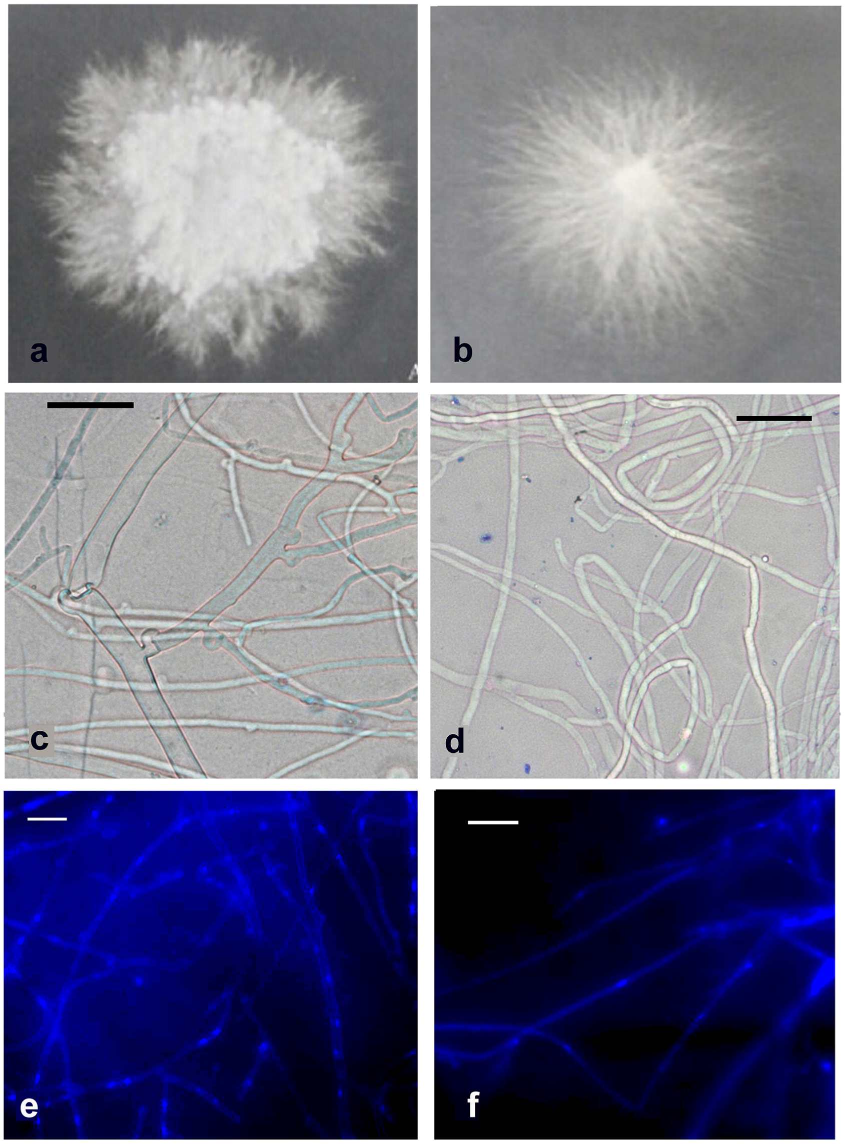

Figure S1. The morphological characters of *Hericium erinaceus*. **a**. the colony of dikaryotic mycelium on PDA medium; **b**. the colony of monokaryon of *H. erinaceus*; **c.** dikaryotic mycelium with obvious clamp connection; **d**. monokaryotic mycelium without conspicuous clamp structures; **e**. dikaryon mycelium with DAPI dye, showing the two cell nucleus with very close distance; **f**. monokaryotic mycelium with DAPI dye, showing the two cell nucleus with very far distance. Scale bar: 20 μm.

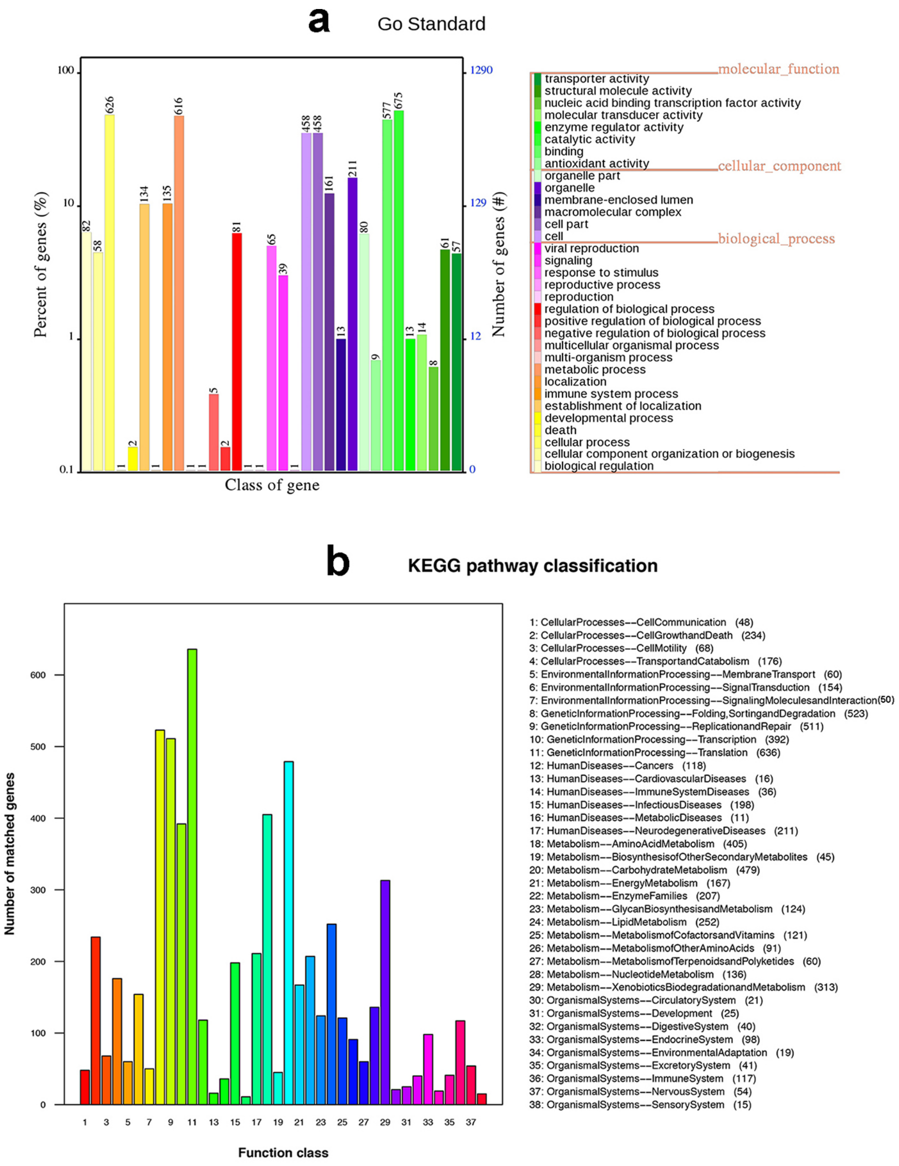

Figure S2. GO and KEGG pathway classification of all annotated 9895 gene models in *Hericium erinaceus* genome (a). All terms belonged to the three main GO categories: biological process, cellular component and molecular function. (**b**). KEGG annotations of all genes, showing the highest number of genes related to metabolism process and carbohydrate metabolism except for genetic information processing.

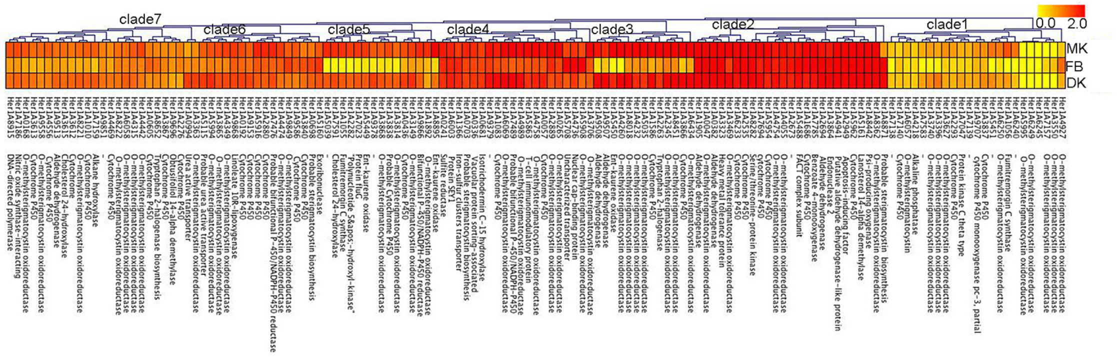

Figure S3. CYPs gene expression across three different tissues of *Hericium erinaceus*. Genesis 1.7.7 software was used for the hierarchical clustering, Log10 (FPKM+1) transformed data were subjected the heatmap. Each tissue is represented by a row of colored boxes (corresponding to REI values) and a single column represents each gene. Regulation levels range from yellow to red (red means high expression and yellow means low expression). Abbreviation: MK, monokayotic mycelium; DK, diokayotic mycelium; FB, Fruiting body.

Table S1. Summary of genome feature of *H. erinaceus*

Table S2. Summary of functional annotation of the predicted genes of *H. erinaceus.*

Abbreviation: Nr, Non-redundant database; KEGG, Kyoto Encyclopedia of Genes and Genomes; KOG, Eukaryotic orthologous groups database; GO, Gene ontology

Table S3. Tissue-specific expression transcript in three different tissues of *H. erinaceus.* A transcript was deemed as tissue specific if the transcript level (fpkm) in this tissue was at least 10-fold higher than in the two other tissues. Expression ratios higher than

100 are colored in red and expression ratios ranging above 50-100 are colored in light red. Abbreviations: MK, monokaryotic mycelium; DK, dikaryotic mycelium; FB, fruiting body.

Table S4. The accession number of Sesquiterpene synthases (STS) used for phylogenetic analysis of *H. erinaceus* in our study.

Table S5. The detailed information of gene clusters predicted by antiSMASH software based on genome of *H. erinaceus*

Table S6. Classification and functional annotation of 137 cytochrome P450s in *H. erinaceus*

Table S7. Genome information used for comparative analysis for gene clusters involved in secondary metabolites biosynthesis in our study.

Table S8. Genes implicated into sexual reproduction of *H. erinaceus*. fpkm value is the mean value of three replicates. Orange color means genes significantly up-regulated and green color means genes significantly down-regulated; gray color means genes no significantly differential expression. Abbreviation: MK: monokaryon mycelium; DK: Dikaryon mycelium; FB: fruiting body. *A. nidulans: Aspergillus nidulan, S. cerevisiae : Saccharomyces cerevisiae* , *S. pombe: Saccharomyces cerevisiae.*
